# Vocal Responses to Perturbations in Voice Auditory Feedback in Individuals with Parkinson's Disease

**DOI:** 10.1371/journal.pone.0033629

**Published:** 2012-03-20

**Authors:** Hanjun Liu, Emily Q. Wang, Leo Verhagen Metman, Charles R. Larson

**Affiliations:** 1 Department of Rehabilitation Medicine, The First Affiliated Hospital, Sun Yat-sen University Guangzhou, People's Republic of China; 2 Departments of Communication Disorders and Sciences, and Otolaryngology, Rush University Medical Center, Chicago, Illinois, United States of America; 3 Department of Neurological Sciences, Rush University Medical Center, Chicago, Illinois, United States of America; 4 Department of Communication Science and Disorders, Northwestern University, Evanston, Illinois, United States of America; Hotchkiss Brain Institute, University of Calgary, Canada

## Abstract

**Background:**

One of the most common symptoms of speech deficits in individuals with Parkinson's disease (PD) is significantly reduced vocal loudness and pitch range. The present study investigated whether abnormal vocalizations in individuals with PD are related to sensory processing of voice auditory feedback. Perturbations in loudness or pitch of voice auditory feedback are known to elicit short latency, compensatory responses in voice amplitude or fundamental frequency.

**Methodology/Principal Findings:**

Twelve individuals with Parkinson's disease and 13 age- and sex- matched healthy control subjects sustained a vowel sound (/α/) and received unexpected, brief (200 ms) perturbations in voice loudness (±3 or 6 dB) or pitch (±100 cents) auditory feedback. Results showed that, while all subjects produced compensatory responses in their voice amplitude or fundamental frequency, individuals with PD exhibited larger response magnitudes than the control subjects. Furthermore, for loudness-shifted feedback, upward stimuli resulted in shorter response latencies than downward stimuli in the control subjects but not in individuals with PD.

**Conclusions/Significance:**

The larger response magnitudes in individuals with PD compared with the control subjects suggest that processing of voice auditory feedback is abnormal in PD. Although the precise mechanisms of the voice feedback processing are unknown, results of this study suggest that abnormal voice control in individuals with PD may be related to dysfunctional mechanisms of error detection or correction in sensory feedback processing.

## Introduction

Idiopathic Parkinson's disease (PD) is a progressive neurodegenerative disease primarily associated with loss of dopaminergic neurons in the pars compacta of the substantia nigra of the basal ganglia. The cardinal symptoms of PD include resting tremor, rigidity, bradykinesia and postural instability [1], [2]. There have also been several studies suggesting that the basal ganglia may be involved in speech, language, stuttering, auditory discrimination, determining basic parameters of movement, error detection, learning behavioral control of movements, and gating of sensory feedback for motor control [3]–[10]. Eighty-nine percent of people with PD have speech and voice disorders that may impair their ability to communicate, characterized by reduced voice amplitude, monotone, breathy, hoarse voice quality, and imprecise articulation [11]. Investigations of vocal output in PD have also reported abnormal perceptual processing of voice and speech auditory feedback [12]–[15] suggesting that input to the speech feedback mechanisms is abnormal, which may explain why some individuals with PD fail to recognize that their voice is not loud enough to be heard by others [12]. Thus, it is also reasonable to conjecture that speech and voice disorders in individuals with PD might be associated with abnormal processing of voice auditory feedback.

One theory on the cause of the movement disorders in PD suggests they may arise from abnormal processing of sensory stimulation (kinesthetic, proprioceptive, auditory) at the cortical and subcortical levels [16]. Other studies have reported that the sensory-triggered reflexes (e.g., perioral, stapedial, stretch), in individuals with PD have greater magnitudes or a lower threshold than in control subjects [10], [17]–[19]. Although there may be other possible neural mechanisms that could account for these changes in functions, evidence has shown that the abnormal status of these functions could be mainly due to the impairment of the basal ganglia [20]. Therefore, impairment of the basal ganglia may cause a loss or error of filtering non-relevant sensory information [21] for movement control, making it difficult to recognize the amplitude of movement or in executing voluntary movements [19]. The basal ganglia may also compare incoming sensory data with motor control networks in the cerebral cortex. In the event of a mismatch, incorrect networks will be activated resulting in abnormal movement patterns [22]. Given the importance of the basal ganglia in sensory processing and motor control, voice and speech disorders in individuals with PD could also be related to the sensorimotor integration deficits arising from dysfunction of the basal ganglia.

Recently, neural mechanisms of the role of auditory feedback on voice control have been studied extensively in healthy adults during both sustained vowels and speech production [23]–[33]. These studies have shown that perturbations in voice pitch or loudness feedback lead to compensatory changes in voice fundamental frequency (F_0_) or amplitude as well as event-related potentials in the auditory cortex [34]–[37]. The mechanisms underlying these responses have been described as a negative feedback control system wherein an efference copy of the intended vocal output is compared with the actual sensory feedback [28]. With a perturbation in auditory feedback, the resulting error from the comparison process generates a compensatory response with a latency of about 100 ms. The responses are thought to be reflexive because of their short latency and because subjects produce the responses without intent [24], [28], [38]. Although the magnitude of the responses is relatively small compared to the size of the feedback perturbation, it is believed voluntary responses that follow the reflexive responses are capable of completing compensation for the perturbation [28].

The present study was designed to test the hypothesis that abnormal processing of voice auditory feedback contributes to the voice disorders observed in individuals with PD who are in an advanced stage of the disease and off medications. The voice perturbation paradigm was used for this testing because it measures the vocal motor responses to perturbations in the acoustical properties of the voice, and relies at least in part on cortical mechanisms. Also, because it was previously shown that individuals with PD have a reduced sense of awareness of laryngeal somatosensory stimulation leading to voice problems [39], and that anesthesia of the laryngeal mucosa leads to larger than normal responses to pitch-shifted feedback [40], it was predicted that individuals with PD would show larger responses to perturbations in voice auditory feedback than healthy control subjects. In the present study,we tested this hypothesis. We examined responses to perturbations in voice loudness and pitch feedback in healthy control subjects and individuals with PD who were off medication. Results confirmed our predictions that the individuals with PD displayed larger response magnitudes than the control subjects.

## Methods

### Ethics statement

Signed informed consented was obtained from all subjects in this study. PD subjects were tested at Rush University Medical Center. Healthy controls were tested at Northwestern University. The test protocol was approved by the Institutional Review Board (IRB) from each institution.

### Subjects

Twelve individuals (10 males and 2 females) with a clinical diagnosis of idiopathic PD and speech impairment participated in the study. The mean age at the time of testing for the PD group was 62.25 (SD 10.15) years, ranging from 45 to 75 years (Table 1). The mean disease duration was 10.58 (SD 4.76) years. The subjects' performance on non-speech motor tasks was rated by the third author, LV, a movement disorders neurologist, using the motor section of the Unified Parkinson's Disease Rating Scale (UPDRS-III) [41]. The mean UPDRS-III motor score was 36.88 (SD 15.12) in the off-medication state, ranging from 18 to 58.5 with a total number of possible points of 108. The mean Hoehn & Yahr [42] stage was 3.14 (SD 0.90) in the off-medication state, ranging from 2 to 4. All had functional hearing and without dementia. Two subjects (S8, S10) had been diagnosed with mild depression associated with PD and both were treated with Celexa. Subject 2 was treated with Amitriphyline for sleep disorder. All subjects were tested in the off-medication state (12 hours off anti-Parkinsonian medication). For the three subjects who had previously received unilateral deep brain stimulation (DBS) of the subthalamic nucleus (STN), the stimulator was turned off for 12 hours before the testing. Clinical descriptions of speech and voice characteristics were provided by the second author, E.W., a licensed speech language pathologist who is specialized in working with individuals with PD and speech impairment.

**Table 1:** PD Subjects Information.

Subject #	Sex	Age at Testing	Disease Duration	Hoehn & Yahr Stage Off-Meds	UPDRS-III Midline Scores Off-Meds	UPDRS-III Motor Scores Off-Meds	UPDRS #18 Speech	Speech Impair.*	Previous surgery for unilateral STN DBS
S1	M	64	10	3	12	35	2	Mild hypophonic	
S2	M	71	19	4	21.5	58	0	Mild drooling	
S3	M	55	5	4	9	31.5	0	Mild-moderate dysarthric	Yes
S4	M	45	8	4	13	32	1	Mild dysarthric	
S5	F	73	8	4	15.5	37.5	1.5	Mild dysarthric	
S6	M	69	3	2	7	28	0.5	Mild hypophonic	
S7	M	60	15	3	10.5	40	1	Mild dysarthric	
S8	M	75	11	3	21.5	58.5	2	Mild-moderate vocal tremor, hoarseness	Yes
S9	M	52	10	2	15	52.5	1.5	Mild dysarthric, drooling on left side	Yes
S10	M	50	10	2.5	8	18	0	Mild hypophonic	
S11	M	73	18	4	15.5	49.5	2	Moderate vocal tremor, moderate dysarthric	
S12	F	60	10	2	6	22	0	Mild vocal tremor, mild hypophonic	
**Mean**	**M**	**62.25**	**10.58**	**3.14**	**12.88**	**36.88**	**0.96**		
**STD**		**10.15**	**4.76**	**0.90**	**5.17**	**15.12**	**1.00**		

* hypophonic: impairment limited to soft voice; dysarthric: impairment involved articulation as well.

The 13 control subjects (11 males and 2 females) had a mean age of 68.7 years (SD 10.6) ranging from 49 to 89 years. Both testing sites used identical equipment, and the PD subjects were tested in a quiet office environment (ambient noise level was about 50 dB SPL). In a previous study it was determined that absolute control of voice auditory feedback or added noise had no effect on vocal responses to pitch-shifted feedback, and therefore it was considered that the low ambient noise level of the office environment would not affect the results in the present study [24]. Control subjects had no known cognitive, speech, and hearing problems and were free of any neurological problems at the time of the testing. All subjects passed a hearing screening at 250, and 500 Hz at 25 dB HL; and 1000–8000 Hz at 50 dB HL. It is unlikely that differences in hearing threshold would have affected the results since it was previously reported that 10 to 20 dB SPL changes in voice amplitude feedback, with or without added noise do not affect the responses (Burnett et al., 1998). Statistical analyses showed no significant difference in the age between PD and control groups (t = −1.450, p = 0.161).

### Procedures

The same instrument set up and equipment were used for testing for both subjects with PD and control subjects. While seated in the examination room, the subjects' voices were recorded with an AKG boom-set headphone and attached microphone (model K270 H/C) with a mouth-to-microphone distance of 1 inch, and amplified with a MOTU Ultralite (model 8410) firewire audio interface, and shifted in pitch or loudness with an Eventide Eclipse Harmonizer. MIDI software (Max/MSP v.4.1 by Cycling 74) was used to control the harmonizer. A Brüel & Kjær sound level meter (model 2250) was used to calibrate the microphone and headphones to a set gain of 10 dB SPL between the subject's voice amplitude and the feedback loudness. The voice output, feedback and TTL control pulses were digitized at 10 kHz by PowerLab (AD Instruments), and recorded using Chart software (AD Instruments).

Each subject was tested under three conditions, one pitch-shift and two loudness shift conditions. For each condition, subjects produced 8–13 vocalizations (sustained vowel /α/) for approximately 5 sec. During each vocalization, either the subject's voice loudness feedback was shifted upwards or downwards by 3 dB or 6 dB (200 ms duration) or voice pitch was shifted up or down by 100 cents (100 cents equals one semitone). Most subjects received five shifts per vocalization over eight vocalizations, resulting in 40 shifts per block of trials (20 upward and 20 downward). However, some of the PD subjects were unable to hold their vocalizations for 5-sec durations, and in these cases three shifts were delivered for each vocalization, and the subjects vocalized 12–13 times, resulting in about 36 to 39 shifts per block (18 to 19 shifts in each direction). In a previous study we determined that varying the number of perturbations per trial from 1 to 10 results in no difference in response magnitudes [43]. During each trial, the timing and direction of the shift was randomized (500–700 ms inter-stimulus intervals).

### Data analysis

Digitized voice and feedback signals were analyzed using an event-related averaging technique offline in a lab computer. For the measurement of vocal response during the perturbed loudness feedback, the voice signal was converted to a root-mean-square (RMS) voltage signal using Igor PRO software (v.6.0, Wavemetrics, Inc.). A RMS wave was obtained using a 50 ms sliding window:
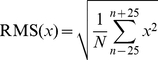
(1)where 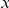 is the value of each data point, and 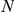 is the total number of data points. Voice RMS voltage measures were then converted to dB SPL to reflect actual voice amplitude using the following formula:
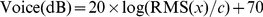
(2)where 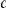 equaled 0.228, which is the RMS voltage corresponding to a vocal level of 70 dB SPL that was obtained through calibration procedures.

For the measurement of vocal responses during perturbed pitch feedback, the voice signal was processed by Praat [44] using an autocorrelation method to produce a train of pulses corresponding to the fundamental period of the voice waveform. This pulse train was transformed to an F_0_ contour wave in Igor PRO, and then converted to a cent scale using the following:
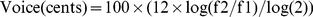
(3)where 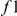 is an arbitrary reference note at 195.997 Hz (G4), and 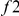 is the voice F_0_ in Hertz.

The voice F_0_ or RMS signal of all trials per block for each subject was then time aligned to the TTL pulses (stimulus onset), and averaged responses to upward and downward stimuli were calculated. Initial acceptance of averaged vocal responses was defined by the averaged waveform exceeding a value of two standard deviations (SDs) of the pre-stimulus mean beginning at least 60 ms after the stimulus and lasting at least 50 ms [25]. The criterion for the end of a response was that it returned to within two SDs for at least 30 ms. Latency of the averaged response was defined as the time from the stimulus onset at which the response exceeded 2 SDs of the pre-stimulus mean. Response magnitude was defined as the maximum deviation of F_0_ from the baseline. Response magnitude and latency values were submitted to significance testing using repeated-measures ANOVAs (SPSS, v. 16.0). There were 3 cases (see Table 2) in which a F_0_ or amplitude response could not be measured. In theses cases, for statistical analysis, the missing value was replaced by the mean of the values from other subjects within the same condition. This is a standard procedure that is routinely used to meet the assumption of the repeated-measures ANOVA [45]. Assumptions of compound symmetry and circularity for repeated measures ANOVA were met. An alpha level of p<0.05 was considered to be statistically significant.

**Table 2:** Numbers of responses (Following, Non-Responses, Opposing) to the loudness- and pitch-shift stimuli for each group of participants (CT – Control; PD – Parkinson).

	CT-LOUD	CT-PITCH	PD-LOUD	PD-PITCH	TOTAL
FOLLOWING	2	0	1	1	4
NR	0	0	2	1	3
OPPOSING	50	26	45	20	141
TOTAL	52	26	48	22	148

## Results

Table 2 provides the number of each response type for each group of subjects, including the pitch- and loudness-shift conditions and the type of response. The responses were separated into three types: opposing (response and stimulus change in the opposite direction), following (response change was in the same direction as the stimulus), and non-response (response that fails to reach the criteria of valid response). Ninety-five percent of responses opposed the stimulus direction, while only four followed the direction of the stimulus. In addition, there were three instances where PD subjects did not register a valid response.

**Figure 1:**
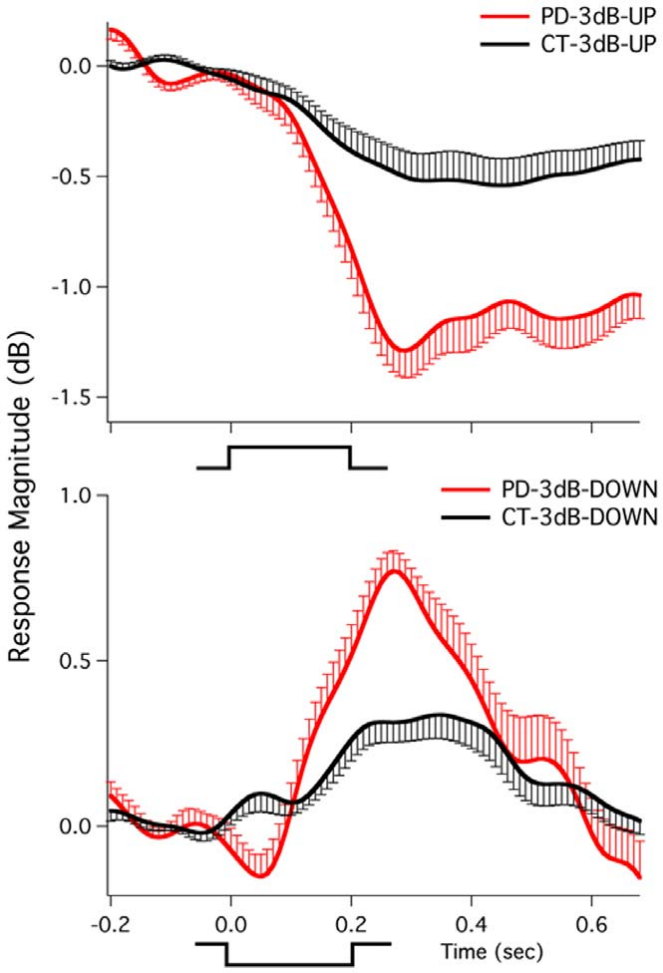
Grand-averaged vocal responses to upward (top) and downward (bottom) 3 dB perturbations across all PD and control subjects. The red and black lines denote the averaged responses produced by the PD and the control subjects, respectively. Vertical bars represent the standard errors of averaged traces. Stimulus timing and direction are illustrated by square trace at bottom of panels.

**Figure 2:**
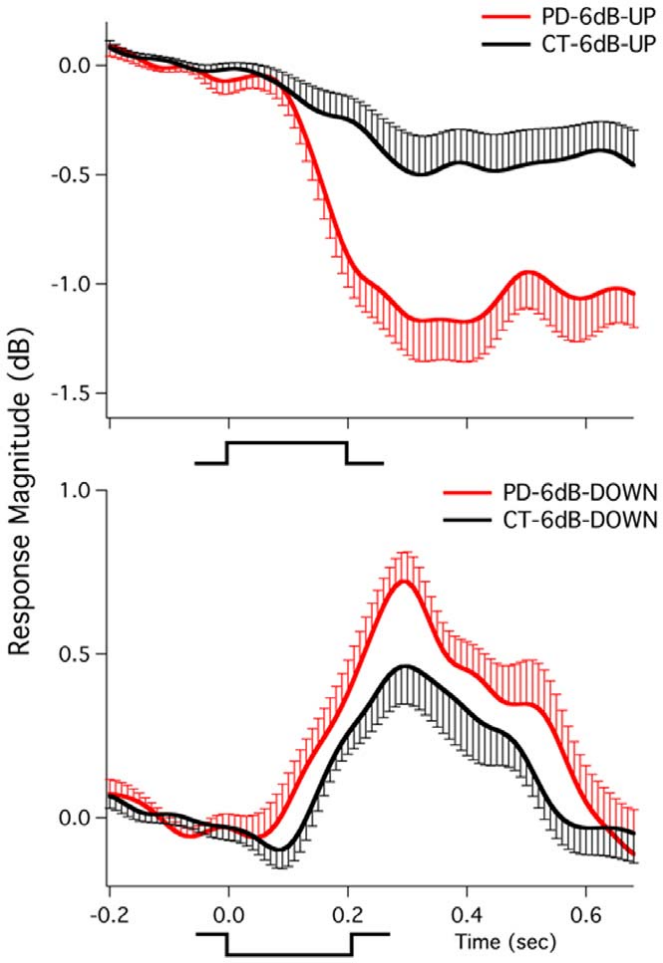
Grand-averaged vocal responses to upward (top) and downward (bottom) 6 dB perturbations across all PD and control subjects. See Figure 1 for details.

**Figure 3:**
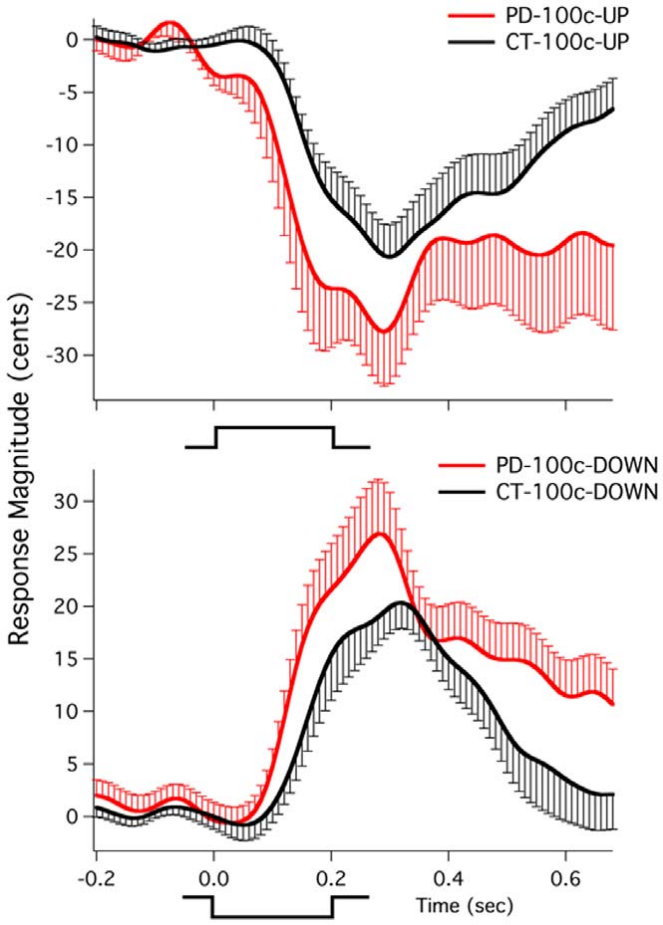
Grand-averaged vocal responses to upward (top) and downward (bottom) 100 cents perturbations across all PD and control subjects. See Figure 1 for details.

Figures 1, 2, and 3 show grand-averaged vocal responses to 3 dB, 6 dB, and 100 cents perturbations in auditory feedback across all PD and control subjects. As shown in Figures 1 and 2, both PD and control subjects produced compensatory voice amplitude responses in the direction opposite to the perturbation in voice loudness feedback. In addition, the PD subjects produced larger vocal response magnitudes in comparison with the control subjects, especially for responses to upward perturbations. Similarly, the PD subjects showed larger response magnitudes to pitch perturbations than the control subjects (Figure 3).

**Figure 4:**
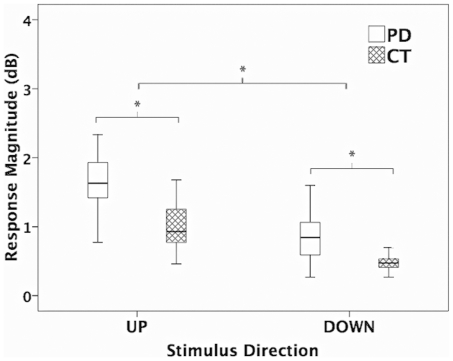
Box plots illustrating the voice loudness response magnitudes as a function of stimulus direction and testing group. Box definitions: middle line is median, top and bottom of boxes are 75th and 25th percentiles. Cross-hatched boxes and blank boxes represent the responses for the control group and the PD group, respectively. The asterisks indicate significant differences between conditions.

Voice amplitude response magnitudes were tested in a three-way (testing group, stimulus magnitude, stimulus direction) repeated-measures ANOVA, which revealed significant main effects for testing group (F(1, 23) = 25.543, p<0.001) and stimulus direction (F(1, 23) = 84.536, p<0.001) but not for stimulus magnitude (F(1, 23) = 1.133, p = 0.298). The PD group produced significantly larger response magnitudes (1.31±0.80 dB) than the control subjects (0.74±0.35 dB), and upward stimuli were associated with larger response magnitudes (1.36±0.75 dB) compared to downward stimuli (0.67±0.32 dB) (see Figure 4).

**Figure 5:**
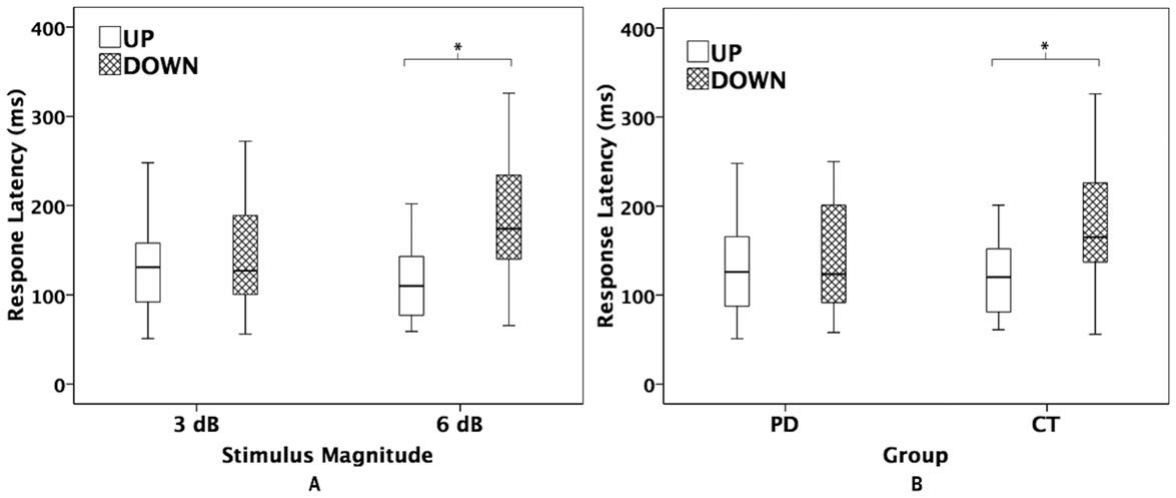
Box plots illustrating the effect of stimulus direction on the voice loudness response latencies as a function of stimulus magnitude (A) and testing group (B). Cross-hatched boxes and blank boxes represent the responses to downward and upward loudness-shift stimuli, respectively. The asterisks indicate significant differences between conditions.

Voice amplitude latencies were also tested in a three-way ANOVA and showed a significant main effect for stimulus direction (F(1, 23) = 8.190, p = 0.009) but not for testing group (F(1, 23) = 1.609, p = 0.217) or stimulus magnitude (F(1, 23) = 0.588, p = 0.451). A significant interaction was found between stimulus direction and stimulus magnitude (F(1, 23) = 10.530, p = 0.004). Further statistical analyses showed significantly longer latencies to downward stimuli (186±71 ms) than upward stimuli (121±56 ms) for 6 dB stimuli (F(1, 23) = 16.264, p = 0.001) but not for 3 dB stimuli (147±71 ms vs. 140±56 ms) (F(1, 23) = 0.159, p = 0.694) (see Figure 5A). In addition, directional effects on the voice amplitude latencies were observed in the control group (F(1, 12) = 12.457, p = 0.004), where downward stimuli led to significantly longer latencies (187±76 ms) than upward stimuli (129±60 ms), but they were absent in the PD group (145±64 ms vs. 132±54 ms) (F(1, 11) = 0.485, p = 0.501) (see Figure 5B).

**Figure 6:**
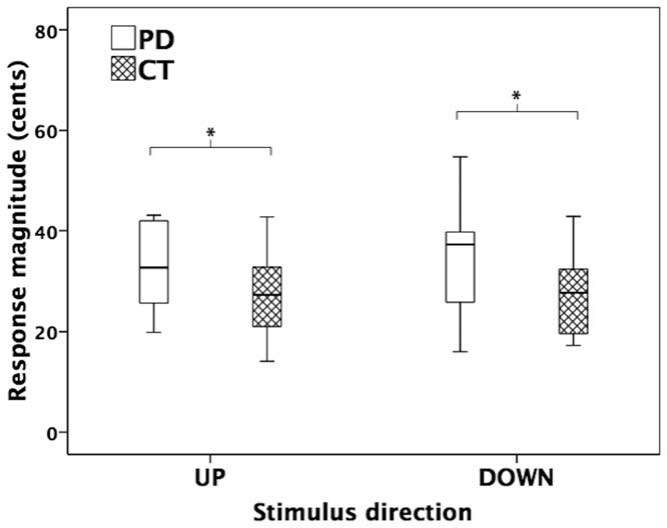
Box plots illustrating the voice F_0_ response magnitudes as a function of stimulus direction for the control group (cross-hatched boxes) and the PD group (blank boxes). The asterisks indicate significant differences between conditions.

Testing voice F_0_ response magnitudes in a two-way (testing group, stimulus direction) repeated-measures ANOVA indicated that the PD group produced significantly larger vocal response magnitudes (37±17 cents) than the control group (27±9 cents) (F(1, 22) = 4.715, p = 0.041) (see Figure 6), but no significant differences were found for stimulus direction (F(1, 22) = 1.172, p = 0.291) or for interactions between testing group and stimulus direction (F(1, 23) = 0.894, p = 0.355) (The pitch-shift reflex was not tested in one of the PD subjects due to time limitations.). For the response latency, neither significant main effects nor significant interactions were found across all conditions.

## Discussion

One of the most significant features of the speech of individuals with PD is that their voices are not loud enough, which interferes with their ability to converse with others. Although the control of voice loudness appears to be a greater problem for communication in individuals with PD than does the control of voice F_0_, a monotonous voice is also a recognized symptom of individuals with PD [11]. Results from the present study showed that individuals with PD produced significantly larger vocal response magnitudes to perturbations in voice amplitude and pitch feedback when compared with age- and sex-matched control subjects. These findings suggest that individuals with PD may have perceived or processed the pitch- and loudness-shift stimuli differently than did the control subjects.

In a previous study on normal subjects using the same techniques as the present study, Bauer et al. [23] reported that the amplitude of loudness-shift reflex was larger when people vocalized with a soft voice amplitude compared to a normal voice amplitude. Thus, it may be argued that larger loudness-shift reflexes for individuals with PD in the present study resulted from their softer voice amplitude as compared to the healthy controls. However, variability in voice amplitude served as a within-subject factor in Bauer et al.'s study [23], and so far there is no direct evidence to support the conclusion that this would also be true between groups of subjects. Moreover, it has been reported that voice F_0_ level is not related to measures of the pitch-shift reflex within different groups [46]. Clearly, these issues should be investigated further. Unfortunately, due to the limitations in the experimental design, the data from the present study do not provide direct evidence to support the suggestion that differing voice loudness levels between groups of subjects would have led to reflexes of different magnitudes observed.

In addition to abnormal response magnitudes, the timing of the vocal responses to perturbed auditory feedback appeared to be different in the individuals with PD compared to the healthy controls. Specifically, in the control group, latencies of voice amplitude responses to upward perturbations were shorter than those to downward perturbations, but similar differences were not observed in the PD group. Such longer latencies to downward voice loudness perturbations compared with upward perturbations were also reported in a group of young healthy subjects [47]. A possible explanation of this finding is that an increase in voice loudness may be more perceptive than a decrease in loudness, which may have triggered a more rapid response. It was also previously shown that timing of voice F_0_ responses to voice pitch-shifted feedback were different in PD subjects compared with healthy control subjects [48]. Taken together, these findings suggest that individuals with PD may differ from the healthy controls not only in the magnitude but also in the timing of vocal responses to perturbations in voice auditory feedback.

Finally, it was observed that in both subject groups, response magnitudes to increases in loudness-shifted feedback were larger than those to decreases in loudness-shifted feedback. Similar findings were previously reported [23], [47], in which normal subjects were presented with perturbations in pitch, loudness and combinations of pitch and loudness feedback. The most likely explanation for these observations is that equal magnitude increases in the loudness of sounds are more perceptible than equal magnitude decreases in loudness [49]. However, since both subject groups showed larger response magnitudes to increases in loudness-shifted feedback compared with decreases in feedback, this responses tendency does not appear to be affected by Parkinson's disease.

An interesting group of studies bears directly on the present study from the standpoint of altered auditory feedback and voice control. The Lombard effect demonstrates that when speakers are presented with loud masking noise, they increase their voice loudness as part of a compensatory mechanism [50]. This technique has been shown to increase voice loudness in individuals with PD [51], [52]. The corollary to the Lombard effect is that of side-tone amplification. In the latter, a speaker responds to an increase in voice feedback loudness by reducing voice amplitude, and to decreases in voice feedback loudness with an increase in voice amplitude. This phenomenon is thought to represent the same mechanisms as the Lombard effect [50]. In both of these conditions, the speaker attempts to increase communicative effectiveness in the presence of competing auditory stimulation. However, given the long temporal nature of the stimulus durations in the above studies [51], [52], it is likely that the responses reflect both reflexive and voluntary mechanisms. In the present study, the stimuli were 200 ms in duration, which have previously been shown to elicit reflexive responses [24], [28]. Therefore, the increased voice amplitude and F_0_ responses to reductions in voice loudness or pitch feedback associated with individuals with PD in the present study, show that the reflexive components of the Lombard and side-tone amplification effects are impaired in individuals with PD, but the present study cannot rule out the possibility that voluntary mechanisms of vocal control are also affected in PD.

The voice auditory feedback perturbation paradigm has proven to be very important for the understanding of how the nervous system responds to changes in auditory feedback for vocal control. Hain et al. [28] proposed an internal model of the audio-vocal system to process the F_0_ drive signal from vocal motor cortex, suggesting that auditory feedback works as a negative feedback, closed-loop system during vocalization. This model was also demonstrated to be a feasible representation of internal circuitry that produces vocal responses to loudness perturbations [23]. In this model, auditory input (auditory feedback) is compared with the efference copy of F_0_ or voice amplitude (the intended feedback), generating a perceptual pitch or loudness error signal. This error signal is further processed at the level of the cortex and brainstem, leading to a final vocal output, which reflects the correction in the response to pitch or loudness perturbations in auditory feedback.

Given this model of processes that may be involved in control of the voice, it is constructive to consider research on the basal ganglia and the role of sensory feedback in motor control. Animal studies have demonstrated that the basal ganglia play a crucial role in error detection, indicating that dopaminergic neurons in the basal ganglia can produce phasic activity projected to other parts of the brain in order to modulate future behavior when an error in the prediction of a future salient event occurs [53]. Falkenstein et al. [13] reported that the impairment of the basal ganglia in individuals with PD causes deficits in error detection in comparison with normal healthy people. Further evidence has demonstrated that the basal ganglia are involved in the sensory processing of auditory information [54], [55]. Due to PD, the basal ganglia may no longer be able to effectively filter relevant from non-relevant sensory information [21], resulting in an error in perception. In the present study, therefore, the impairment of the basal ganglia in individuals with PD may have caused an error in the detection of the magnitude of loudness or pitch changes, which could in turn lead to an error in the correct scaling of the responses. These impairments could then lead to the greater response magnitudes to auditory feedback perturbations than in the control participants.

The impairment of error detection and/or correction in individuals with PD may be related to abnormal sensorimotor integration, possibly involving reduced efference copy of the motor output. The efference copy is thought to predict the sensory consequences of the actions [56], [57]. Sensory processing mechanisms rely on a subtractive comparison of the efference copy with the actual sensory feedback [58], [59], and if there is an error resulting from this comparison, a corrective response is produced [60]. It was suggested from studies in the limb literature that the efference copy may be reduced in individuals with PD such that the error signal resulting from a subtractive comparison between motor output and sensory feedback is reduced, which could result in the classic reduction in output [hypokinesia; [61]] and other abnormalities such as drooling and tremor. Rickards and Cody [19] have argued that the core deficit in PD is a reduction in the efference copy resulting in reduced overall output, which in speech would relate to the monotone and reduced loudness of the voice. That is to say, a reduced efference copy means that the subject thinks he or she is producing a response of adequate magnitude, but the actual magnitude perceived by others is too low. With a deficit in the efference copy mechanisms, the comparison with the sensory feedback would be inaccurate, which could lead to the larger responses reported here.

There are other ways in which abnormalities in sensorimotor integration could possibly lead to the enhanced responses we report. The present study manipulated auditory feedback, however, there are other forms of sensation that could affect vocal control, and the neural processing of them, or the interactions with other forms of sensation and auditory feedback could affect the responses to the pitch- or loudness-shifted feedback. For example, Hammer and Barlow [39] reported that individuals with PD had higher thresholds for detecting somatosensory stimulation of the laryngeal mucosa than did healthy control subjects. The individuals with PD also had reduced subglottal air pressure, air flow rates, laryngeal resistance and lung air volume compared to control subjects. These aerodynamic measures are consistent with reduced voice loudness of individuals with PD. Thus, more severe laryngeal somatosensory deficits in individuals with PD were associated with greater reductions in their voice loudness and monotone quality. Moreover, in a previous study, Larson et al. [40] anesthetized the vocal fold mucosa in healthy subjects and observed that their voice F_0_ responses to pitch-shifted voice feedback were larger than in the absence of anesthesia. These results were interpreted to mean that in the normal situation, both somatosensory and auditory feedback are used to help control voice F_0_. When one of these modes of sensation is reduced or eliminated, the nervous system places greater value on the intact sensation form. Therefore, if individuals with PD have reduced somatosensory input from the larynx, the imbalance between somatosensory and auditory feedback could result in the nervous system placing greater weight on auditory feedback, which could in turn lead to the enhanced responses to the pitch- or loudness-shifted feedback than those in healthy subjects.

Data from other studies more directly link symptoms of PD to the interactions between the basal ganglia and cortical mechanisms. Several transcranial magnetic stimulation and functional imaging studies have shown reduced motor cortex inhibition, or over activity of motor cortical areas in PD [62]–[65], and reduced inhibition of responses was also found in electrophysiological studies [66], [67]. This inhibition may be related to the reduction in suppression resulting from abnormal sensorimotor integration mentioned above. Given that one major function of the basal ganglia is to facilitate the synchronization of cortical activity of a movement [68], these findings suggest that basal ganglia dysfunction may indirectly affect the processing of sensory feedback related to movement control.

Zarate and Zatorre [69] conducted an fMRI study during pitch-shifted auditory feedback in singers and non-singers and found that the anterior cingulate cortex, auditory cortices, and putamen were recruited as people learned to monitor their voice pitch feedback and adjust their vocal responses accordingly. This finding also suggests that the basal ganglia may become involved in the modulation of vocal responses to auditory feedback perturbations (i.e. feedback error correction). The effectiveness or reduction of the inhibition effect on the motor cortex due to the impairment of basal ganglia may lead to the failure of inhibiting or overruling the incorrect/abnormal reflexive response, which accounts for the larger vocal reflex magnitudes to the pitch/loudness feedback perturbations.

These modulatory effects of the basal ganglia on cortical function may help to explain abnormal reflexes in PD reported by others [10], [12], [16], [17], [19], [52], [70]–[76]. Although the precise mechanisms underlying these observations may be similar to the release from cortical inhibition, this is not certain. Nevertheless, all these observations support the general idea that dysfunction in the basal ganglia can impair the modulation of sensory input to cortical or subcortical areas resulting in abnormal reflexes either at the brainstem or cortical level [8], [22], [75], [77].

This is a preliminary study indicating that individuals with PD have larger vocal responses to perturbations in voice loudness- or pitch-shifted voice feedback compared with healthy control subjects. This study provides evidence suggesting that individuals with PD have abnormal sensorimotor integration of voice F_0_ and intensity. It should be pointed out, however, that one primary limitation of the present study is the lack of direct correlational analysis between speech measures and vocal responses. Speech measurements of F_0_ and intensity (e.g. mean intensity, pitch/intensity variability) were not provided for either PD or control groups. As such, it is not possible to determine whether the abnormal voice loudness responses are associated with speech measures in individuals with PD. Thus, the present study does not allow a conclusion about a relationship between the response patterns to perturbed voice loudness feedback and disordered Parkinsonian speech. Future studies could improve on the present one by using electrophysiological, neuroimaging and dynamic causal modeling techniques to help understand neural mechanisms of these responses, testing of a larger cohort of PD participants, and to correlate other measures, such as laryngeal somatosensory sensitivity or specific speech/voice impairments with vocal measures such as the ones in the present study.

### Conclusion

We investigated the vocal responses to perturbations in voice pitch and loudness auditory feedback generated by individuals with PD and control subjects. Individuals with PD produced significantly larger response magnitudes than control subjects to both pitch and loudness-shifted voice auditory feedback. Additionally, larger response magnitudes were found for upward loudness-shift stimuli than for downward stimuli in both of subject groups. These data support the hypothesis that individuals with PD and the control subjects differed in the sensory processing of pitch/loudness errors in voice auditory feedback, suggesting that basal ganglia dysfunction may impair the sensorimotor integration of auditory feedback in individuals with PD.
